# Prevention of intraocular pressure elevation with oleuropein rich diet in rabbits, during the general anaesthesia

**DOI:** 10.1186/s40064-016-2402-3

**Published:** 2016-07-01

**Authors:** Tuncer Şimşek, Uğur Altınışık, İsmail Erşan, Hasan Şahin, Betül Altınışık, Mesut Erbaş, Çiğdem Pala

**Affiliations:** Department of Anesthesiology and Reanimation, Medical Faculty of Çanakkale Onsekiz Mart University, Çanakkale, Turkey; Department of Ophthalmology, Medical Faculty of Çanakkale Onsekiz Mart University, Çanakkale, Turkey; Department of Food Engineering, Engineering Faculty of Çanakkale Onsekiz Mart University, Çanakkale, Turkey

**Keywords:** Oleuropein, Rocuronium, General anaesthesia, Intraocular pressure, Rabbit

## Abstract

**Background:**

Oleuropein is known to have anti-oxidant and anti-inflammatory effects. An important aim of anesthetic management in ocular surgery is to keep the intraocular pressure under control. Studies have researched a variety of prophylactic materials used to prevent increases in intraocular pressure. We aimed to research the effects of oleuropein on intraocular pressure (IOP) during general anaesthesia.

**Methods:**

Fourteen New Zealand rabbits were randomly divided into two groups of seven. The rabbits in Group O were given olive leaf extract (OLE) equivalent to a daily dose of 20 mg/kg oleuropein for 15 days. HPLC method used for oleuropein standardization. For anaesthesia induction 1 mg/kg rocuronium was given and after muscle relaxation all animals had a V-gel Rabbit inserted. Anesthetic maintenance was provided by 1 MAC isoflurane. Twenty minutes after rabbits were given 10 mg/kg ketamine, basal IOP values were measured. After the V-gel rabbit was inserted, in the 5th, 10th, 20th, 25th and 30th minutes measurements were repeated.

**Results:**

IOP data variation of OLE group was compared with control group and the measured levels were lower in Group O during the anaesthesia. IOP was 33.8 ± 4 mmHg in Group C and 24.1 ± 8 mmHg in Group O in 25th minute and the difference between the two groups was statistically significant at this time.

**Conclusion:**

We observed that consumption of prophylactic OLE had a reducing effect on IOP in the period before waking in anaesthesia. We believe it is necessary to investigate the effects of OLE on IOP in broad participation patient groups.

## Background

The olive tree has many significant biological characteristics and contains phenolic materials with oleuropein leading the list of these phenolic compounds (Japon-Lujan et al. 2006). Oleuropein is a material that is found in greatest amounts in the olive fruit at the beginning of growth giving the fruit a bitter taste, and reduces in amount as it is metabolized during maturation (Shasha and Leibowitz 1961). The pharmacological benefits of oleuropein in preventing and protecting from diseases are known. Oleuropein has been shown by a variety of scientific studies to have, in addition to anti-oxidant, antihistaminic, cardioproductive and hypolipidemic effects, anti-inflammatory effects, the effect of inhibiting thrombocyte aggregation, anti-atherogenic effect, anticancerogenic effect, antimicrobic properties against gram-positive and gram-negative bacteria, neuroprotective effect and antiviral effects (Andreadou et al. 2006a; Visioli et al. 1998; Petroni et al. 1995; Visioli and Galli 2001; Hamdi and Castellon 2005; Bisignano and Tomaino 1999; Bazoti et al. 2006; Micol et al. 2005).

For general anaesthesia administration, short-effect anesthetic agents are mostly chosen. These agents provide rapid and reliable recovery, in addition to comfortable anaesthesia (Wandel et al. 1995; Larsen et al. 2000; Loop and Priebe 2000). An important aim of anesthetic management, especially in ocular surgery, is to keep intraocular pressure under control. During and after ocular surgery sudden and large changes in intraocular pressure may cause damage to the vision functions of these patients postoperatively. As a result, it is necessary to protect this patient group from mechanical and pharmacological stress that increases intraocular pressure (Schäfer et al. 2002).

Intraocular pressure (IOP) is defined as the pressure components within the eye apply against the fibrous tunic of the eye. IOP levels are determined by the volume of ocular fluids comprised of the volume of intraocular fluid, volume of choroidal blood and vitreous volume. Ocular compliance (extraocular compression including scleral hardness and extraocular muscle tone) at the same time plays an important role in the regulation of IOP (Qiu et al. 2014). Additionally IOP is affected by changes in blood pressure, central venous pressure, intrathoracic pressure and PaCO2 changes. Apart from this, medications that affect the sympathetic and parasympathetic system, like anesthetics, cause changes in IOP (Camras et al. 2010; Johnson et al. 2008; Lentschener et al. 1998).

Studies have researched a variety of prophylactic materials to prevent increases in intraocular pressure. The reducing effects on intraocular pressure of melatonin, saffron and diosmin, a flavonoid, have been proven (Tong et al. 2012; Martinez-Aguila et al. 2013; Bonyadi et al. 2014). We aimed to research the effects of oleuropein, with anti-oxidant effects like saffron and disomin, on intraocular pressure during general anaesthesia.

## Methods

### Animals

This study was completed in the experimental animals laboratory of Çanakkale 18 Mart University Experimental Research Application and Research Center after receiving permission from the Çanakkale 18 Mart University Animal Experiments Ethics Committee.

The research used 14 adult male New Zealand rabbits. During the experiment animals were fed with standard feed and given continuous access to water, with the living environment temperature set to 21 ± 2 °C with 12 h light and 12 h darkness. Animals were restricted from feeding from 24.00 on the night before the day of anaesthesia.

All the protocols described here comply with the ARVO Statement for the Use of Animals in Ophthalmology and Vision Research and also are in accordance with the European Communities Council Directive (86/609/EEC).

### Study groups

The animals were randomly divided into two groups: oleuropein group (Group O, n:7) and control group (Group C, n:7). Group C were fed with normal water. Group O were fed with water containing oleuropein leaf extract (OLE). 20 mg/kg of Oleuropein was given to rabbits with OLE per day.

### Olive leaf extract (OLE) preparation

OLE preparation made in Çanakkale Onsekiz Mart University Faculty of Engineering Food Engineering Department Laboratories.

Olive leaves were harvested from olive tree (Olea europaea) variety Ladolia grown in Gökçeada (Imbros), Çanakkale, Turkey. Leaves were dried at 80 °C for 4 h. Dried leaves were ground using Delonghi coffee grinder KG49 and then, filtered using 850 mm laboratory sieve. Olive leaf powder was extracted 1:5 (w:v) with hot water in water bath (Memmert WNB 10, Germany) at 80 °C for 10 min. The mixture was filtered using cotton fabric and transferred to falcone tubes (15 mL). Olive leaf extracts (OLEs) were stored at -20 °C to prevent degradation of oleuropein during the experiment.

### Analysis of oleuropein in olive leaf extract (OLE)

A HPLC (High Pressure Liquid Chromatography) assisted method was used for analysis of oleuropein content of OLE (Al-Rimawi 2014). OLE sample was diluted (1:10, v:v) with ultrapure water and filtered through 0.45 μm PTFE syringe filter before injection to HPLC system. 20 µL volume of diluted OLE sample was injected to SHIMADZU LC 20A (Japan) coupled with DAD (Diod Array Detector, SPD M20A). Separation of oleuropein was carried on Inertsil ODS 3 (250 mm, 4.6ID, 5 µ) reverse-phase column under isocratic condition with 80:20 v/v water:acetonitrile (adjusted to pH 3 with acetic acid) as a mobile phase. Column was maintained at 35 °C during chromatographic run. Flow rate was 1 mL/min and the DAD detector was set at 200–400 nm to analyze the spectra. Quantification was made at 280 nm using external standard method. Results were expressed as mg of oleuropein/L of OLE based on seven-point standard curve prepared by the oleuropein standard (Sigma-Aldrich, Germany) solutions range in 10–1000 mg/L concentration.

### Diet protocol

The 7 rabbits in O Group had daily water consumption of 80–100 ml/kg/day calculated according to weight. The amount of oleuropein in OLE was calculated as 4.31 ± 0.18 mg/ml with the amount of OLE found according to rabbit weight for an oleuropein dose of 20 mg/kg. Before feeding plastic tubes containing OLE were dissolved at room temperature and added to drinking water. The consumption of water containing OLE by rabbits was monitored daily. This diet was continued for 15 days.

### General anaesthesia protocol

Before general anaesthesia, all experimental animals were given 10 mg/kg ketamine by i.m. pathway for premedication. With this dose it was aimed that sufficient calming and sedation of rabbits be obtained after 15 min. The injections were administered into the quadriceps femoris muscle. After this procedure, rabbits had arterial and venous catheterization performed in the ear artery and vein with a 26 G catheter and later medications were administered by i.v. For anaesthesia induction 2 mg/kg ketamine and 1 mg/kg rocuronium bromide were administered by i.v. After sufficient muscle relaxation was provided, to ensure airway reliability all animals had a V-gel Rabbit inserted. Pedal and palpebral reflexes were checked to determine whether animals had entered anaesthesia or not. Though the anesthetic duration of animals may show changes, to provide continuity of data in all groups, procedures were completed taking note of a 30 min anesthetic duration.

During general anaesthesia, a pulse oximetry device measured pulse and oxygen saturation in the ear to monitor for reliable anaesthesia. Mean arterial pressure was monitored in the rabbits through arterial cannulization of the opposite ear. Body temperature was measured with a digital thermometer inserted rectally and during anaesthesia body temperature was held at 37–39 °C.

### Achieving mechanical ventilation

All rabbits with paralyzed respiration and V-Gel Rabbit (V-gel rabbit R-3 Docsinnovent^®^ Ltd. London, UK) inserted,to provide reliable airway for all animals and the the animals linked to anesthetic device (Anaesthesia Machine w/O2 Flush Model M3000PK Parkland Scientific Lab And Research Equipment. Florida, USA) were manually ventilated. For maintenance of anaesthesia 1 MAC isoflurane was used. After being linked to the anaesthesia device, respiration count was supported at 40/min and 15 cmH2O pressure, appropriate for rabbit physiology. Ventilation values were set to 50 % oxygen, 50 % air mix with 10 mg/kg volume and inspiration/expiration rate of ½.

### Intraocular pressure measurements

Before general anaesthesia, 20 min after rabbits (Group C and Group O) had premedication with 10 mg/kg ketamine, basal intraocular pressure values were measured. The tonometer was calibrated before the beginning of each measurement and measurements used the same eye of the rabbits. Intraocular pressure measurements were taken and recorded by the same ophthalmologist, who was blinded to experimental procedure. The IOP measurements were taken by using Tonopen-Avia (Reichert Inc., NY, USA) which averages 10 successful readings and displays the mean and SD. Measurements were repeated and recorded in the 5th, 10th, 20th, 25th and 30th minute after the V-gel rabbit was inserted.

### Statistical analyses

Statistical analysis was performed by using the SPSS software version 19 (SPSS 19 for Windows, IBM, Chicago, Illinois, USA). Mann–Whitney U test used to compare the differences between groups. Data were expressed as the mean ± standard deviation. P value less than 0.05 considered as statistically significant.

## Results

The average weight of rabbits in the control group was 3542 ± 170 g while the oleuropein group had an average weight of 3290 ± 438 g. Before the 15 day diet average weight of rabbits in the control group was 3091 ± 120 g and in the control group average weight was 2885 ± 359 g. There was not notable change in rabbit weights before and after the diet. Mean body weights were not statistically different between the groups.

**Fig. 1:**
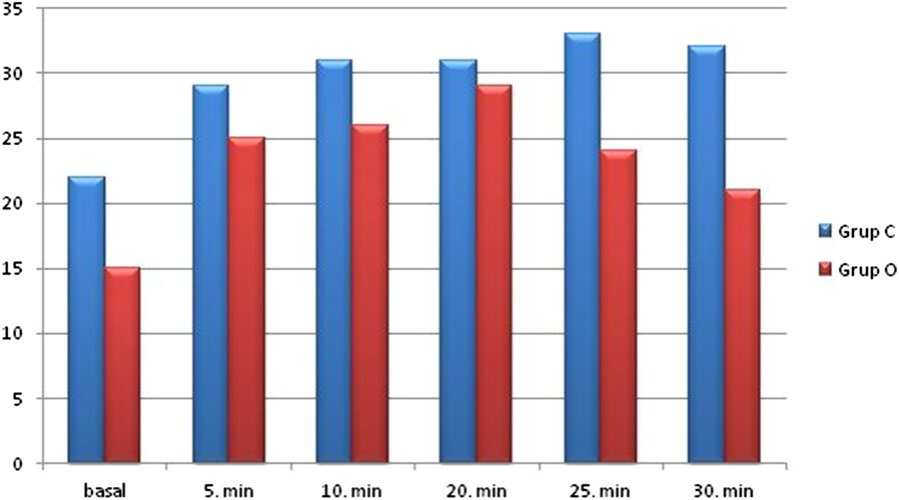
Mean IOP level variation of groups C and O in measurement times during the general anesthesia

Oxygen saturations of animals in both groups remained stable during the course of anaesthesia. The heart rates values from both groups were similar during the experiment (Table 1). In addition there was no significant arterial blood pressure change between the two groups during the anaesthesia. The mean IOP data variation of OLE group was compared with control group, the measured levels were higher in group C during the anaesthesia procedure (Fig. 1).

**Table 1:** Heart rate values in Group C and Group O

HR	Grup C	Grup O	p
0 min	256 ± 48	270 ± 38	>0.05
5 min	246 ± 41	251 ± 46	
10 min	248 ± 43	246 ± 61	
20 min	247 ± 53	236 ± 38	
25 min	239 ± 44	246 ± 29	
30 min	217 ± 40	228 ± 50	

There was no statistically significant difference in both groups

First measurement made 20 min. after the i.m. administration of ketamine. This measurement accepted as baseline (0 min) IOP level. Baseline (0 min) mean IOP measured in control group as 22.2 ± 12 (range 11–50) mmHg. In group O mean IOP was 15.7 ± 5 (range 9–22) mmHg at 0 min. Before the anaesthesia IOP was markedly lower in OLE group but there was no statistically significant difference.

First measurement of anaesthesia procedure made 5 min after the insertion of V-gel rabbit. Mean IOP in both groups was increased to 29 ± 6 (range 15–36) mmHg in group C and 25.5 ± 10 (range 10–34) mmHg in group O 5 min. after the anaesthesia induction.

Mean IOP was increased to 31.1 ± 5 mmHg in group C and 26.4 ± 13 mmHg in group O 10 min. after the anaesthesia induction. IOP difference between the groups was narrowed after the 20 min. of anaesthesia induction. The IOP was 31 ± 5 mmHg in group C nearly the same with the previous measurement. Mean IOP in group O increased at this time and measured as 29.7 ± 8 in group O.

Mean IOP increased to 33.8 ± 4 mmHg in group C and decreased to 24.1 ± 8 mmHg in group O 25 min after the anaesthesia induction. The mean IOP difference between the two groups was statistically significant (p = 0.034) at this time.

Mean IOP was 32.2 ± 7 (range 23–42) in group C and 21.5 ± 10 (range 10–36) in group O 30 min. after the induction of anaesthesia. IOP rates were markedly lower in group O at the end of anaesthesia but there was no statistically significant difference (Table 2).

**Table 2:** Statistical differences between groups calculated by using Mann Whitney U test

IOP (mean)	0 min	5 min	10 min	20 min	25 min	30 min
Group C	22.2 ± 12	29 ± 6	31.1 ± 5	31 ± 5	33.8 ± 4	32.2 ± 7
Group O	15.7 ± 5	25.5 ± 10	26.4 ± 13	29.7 ± 8	24.1 ± 8	21.5 ± 10
p value	0.275	0.654	0.443	0.798	0.034*	0.063

Mean IOP difference was statiscally significant in 25th min of anaesthesia, * p < 0.05

## Discussion

In this study of a general anaesthesia model without surgical stimulus, a prophylactic oleuropein rich diet for 2 weeks was shown to reduce the intraocular pressure in rabbits during and before anaesthesia. This effect was statistically observed to reach significant levels in the 25th minute of the anesthetic procedure.

Intraocular pressure is defined as the pressure of components of the eye against the fibrous tunica of the eye. IOP levels are determined by the ocular fluid volume which included intraocular fluid volume, choroidal blood volume and vitreous volume. Ocular compliance (extraocular compression including scleral hardness and extraocular muscle tone) at the same time plays an important role in the regulation of IOP (Qiu et al. 2014).

During anesthetic procedures IOP control is important. Especially during ocular surgery, an important aim of anesthetic management is keeping intraocular pressure under control. During and after ocular surgery sudden and large changes in intraocular pressure may cause damage to the postoperative vision functions of these patients (Schäfer et al. 2002). Increased IOP disrupts vascular regulation, and additionally applies mechanical pressure to veins supplying the optical nerve and retina, reducing ocular perfusion pressure. All medications used for anaesthesia may change IOP. Also, insertion of endotracheal tube or laryngeal mask airway increases IOP (Alipour et al. 2014). An animal study showing the effect of general anaesthesia on IOP showed that isoflurane lowered IOP and that this effect became more distinct as the duration of anaesthesia lengthened. This study used 20-min duration of anaesthesia and observed that the greatest reduction in IOP was in the 20th minute (Jia et al. 2000). In our study similarly, in the 20th minute the reduction of IOP in the control group was greatest and graphically we observed the difference with the OLE group narrowed at the 20th minute. We believe this situation may have developed linked to the effect of isoflurane increasing with the duration of anaesthesia.

Free radicals released by the cyclooxygenase enzyme pathway within the eye have been shown to disrupt autoregulation of retinal blood flow (RBF) in an animal study. Disrupted autoregulation supports IOP increases and neurodegenerative damage (Hardy et al. 1994). In terms of this mechanism, a variety of prophylactic materials have been researched to prevent intraocular pressure increases. Of these, the reducing effects of curcumin, saffron and diosmin, a flavonoid, have been proven (Tong et al. 2012; Martinez-Aguila et al. 2013; Bonyadi et al. 2014). The common property of these materials is that each is a strong anti-oxidant and some may show anti-inflammatory effects. In fact, though many medications reducing IOP are in use, these medications have the potential for severe side effects. Causing bradycardia and hypertension may be listed among these effects. As their use may be dangerous in patients with cardiac problems, for asthmatics, in those with chronic obstructive pulmonary disease and corneal dystrophy, the choice of a natural material with no side effects becomes important for these patients. As oleuropein has known anti-oxidant, anti-inflammatory and vasodilator effects and no known side effects, it was considered that it might be beneficial for the control of IOP during anaesthesia procedures. There is no previous study reporting the use of oleuropein in this way.

A variety of studies were referenced for the dose of oleuropein for rabbits. Different studies on animals have reported a variety of calculations to determine the dose of oleuropein necessary (Andreadou et al. 2006b; Al-Azzawie 2006). According to these calculations as the anti-oxidant, anti-inflammatory and vasodilator effects are clearly observed with a dose of 20 mg/kg, we chose to use an OLE formulation containing the same dose of oleuropein. Oral tolerance of OLE has been successfully shown. All rabbits in the OLE group consumed water mixed with the extract. A study with mice reported that the intestinal absorption of oleuropein was rapid, reaching peak plasma concentrations in 2 h after a 20 mg/kg oral dose (Del Boccio et al. 2003).

In a general anaesthesia model without surgical stimulus, from the 0 min of basal measurement values before anaesthesia we observed better IOP compared to the control group. This effect continued at other measurement times. A statistically significant difference was observed in the 25th minute of anaesthesia. We link the lack of significance for other measurements to the 25th minute being just before the wakening process and the beginning of the end of the effective duration of intravenous anesthetics in the control group. As a result, the increase in IOP regulation became more distinct in the control group. At other measurement times in the OLE group, IOP values were found to be lower than the control group but we did not reach significant results. The decision to use a limited number of animals in this animal study may have caused this result. We believe studies with larger numbers or participants may find more significant results.

In conclusion; consumption of prophylactic OLE in a general anesthetic process without surgical stimulus showed a reducing effect on IOP especially in the period before induction and wakening. We believe it is necessary to investigate the effects of OLE on IOP in patient groups with broader participation.
